# Cerebrovascular disorders associated with agenesis of the internal carotid artery: Findings on digital subtraction angiography

**DOI:** 10.3389/fsurg.2022.953697

**Published:** 2022-11-07

**Authors:** Xiaolong Xu, Hongjian Shen, Hongyu Ma, Xiaoxi Zhang, Lei Zhang, Qiang Li, Rui Zhao, Dongwei Dai, Zifu Li, Pengfei Yang, Jianmin Liu

**Affiliations:** Neurovascular Center, Changhai Hospital, Naval Medical University, Huangpu, China

**Keywords:** internal carotid artery, agenesis, aneurysm, rete mirabile, dural arteriovenous fistula

## Abstract

**Objective:**

Agenesis of the internal carotid artery (ICA) is a rare vascular condition that is complicated by intracranial aneurysms and rete mirabile. The altered hemodynamics caused by this distinctive cerebrovascular angioarchitecture can cause ischemic or hemorrhagic accidents. Data on clinical and radiographic features have been limited to describing this vascular pattern. We present five cases of agenesis of the internal carotid artery confirmed by digital subtraction angiography (DSA) and further investigate the influence of altered angioarchitecture on the integrity of intracranial morphology.

**Methods:**

Cases of ICA anomalies were screened from the patients who underwent DSA in two hospitals. Clinical manifestation, radiographic features, management, and outcomes were retrospectively reviewed.

**Results:**

Five patients [mean age 44 years (range, 30–65 years)] were included. Two patients presented with subarachnoid hemorrhage, one with cognitive impairment, one with dizziness, and one with intermittent headache. DSA demonstrated that three cases were complicated by intracranial aneurysms, one by dural arteriovenous fistula, and one by rete aneurysm. Three patients underwent endovascular treatment and one underwent bypass surgery. No patient died or experienced cerebrovascular accident during short-term follow-up.

**Conclusions:**

ICA agenesis can be complicated by disorders such as intracranial aneurysm, rete aneurysm, and dural arteriovenous fistula. This suggests that ICA agenesis is associated with a tendency towards disrupted cerebrovascular homeostasis resulting from altered hemodynamics.

## Introduction

Anomalies of the internal carotid artery (ICA), mainly consisting of agenesis, hypoplasia, and aplasia, are rare, occurring in less than 0.01% of the population (1). Some of these anomalies are asymptomatic; others have the potential to cause cerebrovascular accidents or other syndromes (2, 3). The diagnosis of ICA agenesis or hypoplasia relies mainly on the morphology of the ICA and the types of collateral pathways (4). Accordingly, the particular morphological features of absent or hypoplastic carotid canals inform their respective diagnoses. In addition to these features, an enlarged vertebral foramen and hypertrophy of the vertebrobasilar system also provide indirect evidence. More clinical and radiographic data are required to explore the nature of these anomalies. Here we present five cases of ICA anomalies. We aim to investigate the influence of altered angioarchitecture on intracranial morphological integrity.

## Methods

Patients who underwent conventional angiography between January 2010 and December 2013 in two institutions were retrospectively reviewed. Computed tomography angiography (CTA), magnetic resonance angiography (MRA), or digital subtraction angiography (DSA) were used to present the morphology of carotid artery. CT perfusion studies were performed to investigate the intracranial hemodynamics of patients. Clinical manifestations, perfusion characteristics, radiographic features, treatment, and outcomes were analyzed. Radiographic images were downloaded from the picture archiving and communication system and analyzed. Electronic records were reviewed to investigate patients' clinical manifestations, treatment, and outcomes.

The inclusion criteria of ICA anomalies were as following: (1) The absent or hypoplastic internal bony carotid canal revealed by computed tomography. (2) The absence or vascular malformation of the internal carotid artery by CTA, MRA, or DSA (5). Acquired carotid stenosis or occlusion secondary to carotid arteriosclerosis, arteritis, fibromuscular dysplasia, intimal dissection, or Moya-Moya diseases were excluded (2).

A total of five patients were diagnosed with ICA anomalies after screening from 5,850 patients. One patient presented with subarachnoid hemorrhage (SAH), one with cognitive impairment, two with dizziness, and one with intermittent headache. Images from high-resolution computed tomography (CT) or rotational angiography (DynaCT; Siemens Medical Solutions, Erlangen, Germany) showed agenesis of the carotid canal unilaterally in one patient and bilaterally in three patients, and unilateral ICA agenesis with hypoplasia of the contralateral carotid canal in one patient.

## Case reports

### Case 1

**Figure 1:**
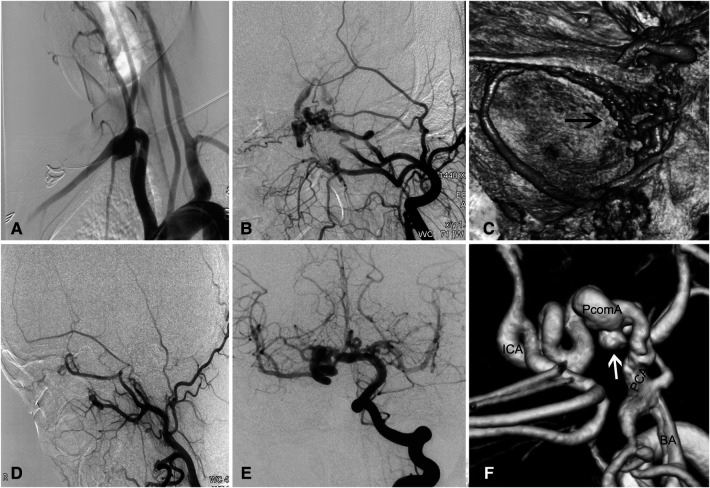
Complicated aneurysm of the posterior communicating artery with bilateral agenesis of the internal carotid artery in a 30-year-old woman. (**A**) Angiogram showing absence of bilateral ICAs. (**B**) Carotid rete mirabile between the external and internal carotid arteries on the left side, where there is ICA agenesis. (**C**) Reconstructed angiogram showing the rete mirabile located around the cavernous sinus. (**D**) No rete mirabile is present on the right side. (**E**) Injection of contrast in the right vertebral artery showing the posterior circulation providing blood supply to the anterior circulation *via* the right posterior communicating artery. (**F**) Reconstructed images showing the dolichoectatic posterior communicating artery harboring an aneurysm (white arrow). BA, basilar artery; ICA, internal carotid artery; PCA, posterior cerebral artery; PcomA, posterior communicating artery.

A 30-year-old woman with intermittent headache underwent CT showing a hyperdense lesion adjacent to the midbrain. High-resolution CT across the petrous bone showed the absence of the carotid canals bilaterally and an enlarged vertebral foramen 8 mm in diameter. DSA and reconstructed images showed absent ICAs bilaterally, a dilated vertebrobasilar system, rete mirabile in the cavernous region, and a dolichoectatic posterior communicating artery (PcomA) harboring an aneurysm (Figure 1). Carotid rete mirabile was not observed on the right side. A CT perfusion scan displayed a prolonged mean transit time (MTT) and time to peak (TTP) and reduced cerebral blood flow (CBF) and cerebral blood volume (CBV) in the bilateral subcortical and border-zone regions. Because the patient was asymptomatic, close angiographic surveillance and control of risk factors were adopted for the patient. The patient's 3-year follow-up was unremarkable.

### Case 2

**Figure 2:**
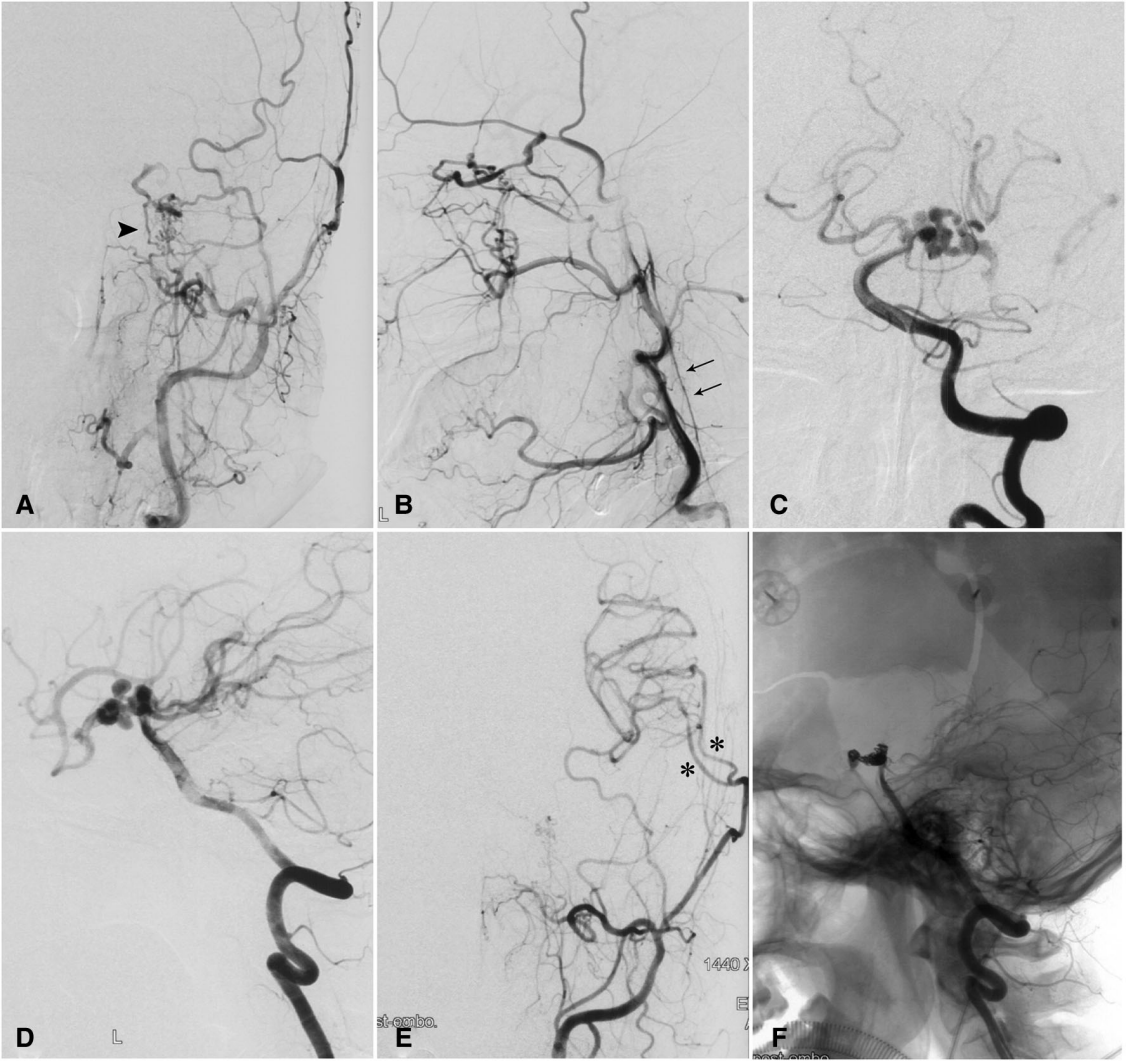
Complicated aneurysm of the posterior communicating artery with hypoplasia of the left internal carotid artery in a 49-year-old woman. (**A**) Injection of contrast in the left common carotid artery showing carotid rete mirabile (arrowhead) between the branches of the external and internal carotid arteries. (**B**) Retrograde flow from an enlarged ophthalmic artery and a hypoplastic left ICA (double arrow). (**C**) Anteroposterior and (**D**) lateral angiograms showing the P1 segment and the posterior communicating artery harboring multiple aneurysms. (**E**) Postoperative angiograms and reconstructed images with anteroposterior and lateral views showing patent bypass arteries (asterisks). (**F**) Coil embolization of aneurysms was performed on an occluded posterior communicating artery. ICA, internal carotid artery.

A 49-year-old woman had SAH confirmed by head CT scan 3 months prior to referral to our institution with presentations of headache, nausea, and vomit. DSA showed hypoplasia of the left ICA and carotid canal, rete mirabile with anastomosis between the ophthalmic and external carotid artery (ECA) branches, and multiple aneurysms of the dolichoectatic PcomA and the P1 segment (Figure 2). A CT perfusion study showed a prolonged TTP and MTT and normal CBV and CBF in the left cerebral hemisphere. Considering the risk of hemodynamic deterioration after endovascular embolization of complicated aneurysms, we first performed double superficial temporal artery-middle cerebral artery bypass surgery. A postoperative CT perfusion scan showed improved hemodynamics in the right temporal region. Nine days later, we performed embolization of the intracranial aneurysms. The patient was discharged without complications, and a 13-month follow-up was unremarkable.

### Case 3

**Figure 3:**
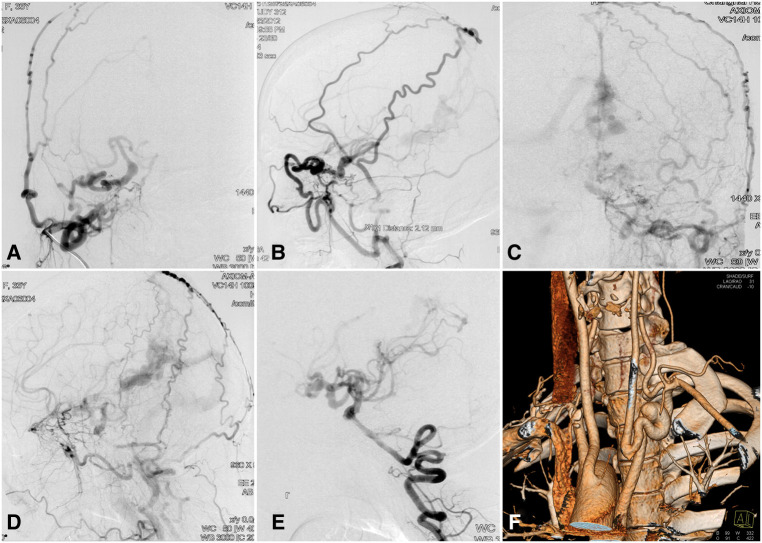
Complicated dural arteriovenous fistula with bilateral ICA agenesis and aortic anomaly in a 33-year-old woman. Contrast injection in the right common carotid artery in (**A**) lateral and (**B**) anteroposterior views showing carotid rete mirabile recruiting the anastomotic vessels between the ICA and ECA branches. Contrast injection of the left common carotid artery in (**C**) lateral and (**D**) anteroposterior views showing a complicated dural arteriovenous fistula in the region of the straight sinus. (**E**) Blood supply of the anterior circulation is provided partly by the posterior circulation *via* the posterior communicating artery. (**F**) CT angiography showing right-sided aortic arch and aberrant left subclavian artery. CT, computed tomography; ECA, external carotid artery; ICA, internal carotid artery.

A 33-year-old woman presented with dizziness, intracranial murmur, and memory decline for 2 months. Her past medical history was unremarkable, and physical examination findings were normal. Magnetic resonance imaging showed a posterior fossa cyst, hypoplasia of the cerebellar vermis, agenesis of the carotid canals bilaterally, and an abnormal flow void around the straight sinus. DSA showed the absence of the carotid canals bilaterally, carotid rete mirabile with collateral anastomosis between the ICA and ECA, and complicated dural arteriovenous fistula (DAVF) fed by the ECA branches and posterior cerebral artery (PCA) (Figure 3). CT angiography demonstrated a right-sided aortic arch with an aberrant left subclavian artery. A CT perfusion scan showed prolonged TTP and MTT and reduced CBF and CBV in the right hemisphere and bilateral subcortical regions. The patient underwent endovascular embolization of the DAVF with Onyx™ Liquid Embolic System 18 (Onyx™ LES; Covidien PLC, Dublin, Ireland). After treatment, the patient reported complete resolution of dizziness and intracranial murmur. Her 2-year follow-up examination was normal.

### Case 4

**Figure 4:**
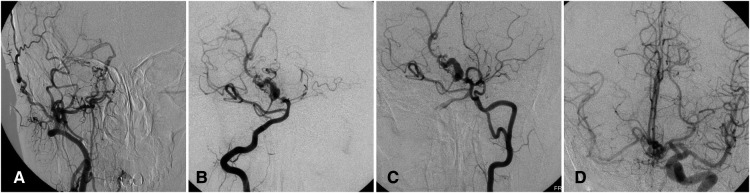
Complicated basilar aneurysm with right ICA agenesis in a 46-year-old woman. (**A**) Angiogram showing carotid mirabile on the right with absent ICA. (**B**) Angiogram *via* right vertebral artery showing basilar aneurysm. (**C**) Angiogram *via* left vertebral artery also demonstrating fenestration of left vertebral artery. (**D**) Angiogram *via* left ICA showing collateral circulation *via* the anterior communicating artery. ICA, internal carotid artery.

A 46-year-old woman presented with a history of recurrent episodes of headache for 40 years. Her history was otherwise unremarkable. CT angiography showed hypoplasia of the right carotid canal and ICA, and aneurysm of basilar artery. DSA further demonstrated rete mirabile formation between the ECA branches and the distal end of the right ICA, and hypoplasia of the basilar artery with aneurysmal dilation (Figure 4). Because the patient's headaches were tolerable, we advised control of risk factors and observation. A 2-year follow-up examination was unremarkable.

### Case 5

**Figure 5:**
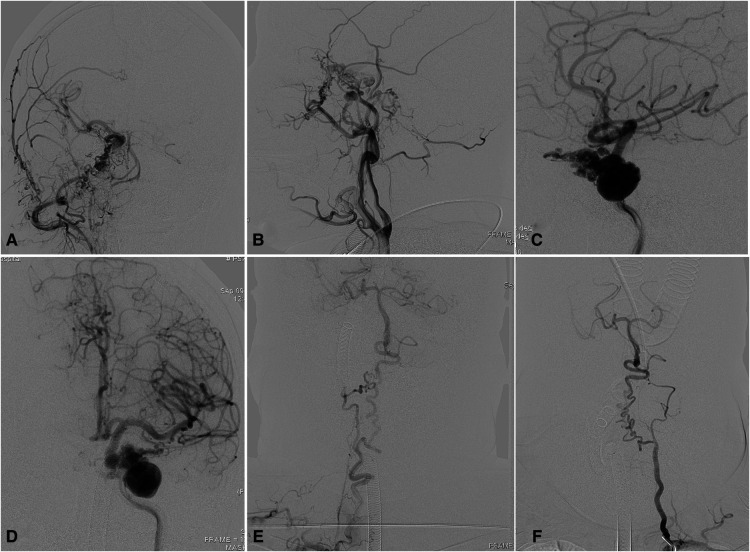
Complicated rete aneurysm with bilateral ICA agenesis in a 65-year-old woman. Angiograms in (**A**) anterior and (**B**) lateral views showing rete mirabile between the ECA branches and distal ICA remnant with right ICA agenesis. Angiograms *via* left ICA in (**C**) anterior and (**D**) lateral views showed that complicated rete aneurysm was developed in the left with segmental ICA agenesis. Elongated and underdeveloped (**E**) right and (**F**) left vertebral arteries. ECA, external carotid artery. ICA, internal carotid artery.

A 65-year-old woman presented with dizziness for 5 days. Magnetic resonance imaging with contrast showed a heterogeneously enhanced mass around the parasellar region. DSA demonstrated dysplasia of bilateral ICAs, a rete aneurysm located in the proximal terminus of the right ICA, and elongated vertebral arteries (Figure 5). Radiographic follow-up was recommended for the patient, and the patient was without symptoms at a 5-month follow-up.

## Results

Clinical and radiographic data for the five patients were listed in Table 1. All the cases presented carotid rete mirabile defined as the arterial plexus between the branches of external carotid artery and ICA. The bilateral hypoplasia or agenesis of ICAs were detected in 3 patients. Six of the 8 ICAs with anomalies developed ipsilateral rete mirabile. The CT perfusion results showed prolonged MTT and TTP in three patients, and reduced CBF and CBV were in two patients.

**Table 1:** Patient characteristics.

Case	Sex	Age (y)	Symptom	Initial detection	ICA anomaly type by DSA	CT perfusion (location/sign)	Location of rete mirabile	Complicated disorder	Management	Follow-up (period/result)
1	Female	30	Headache	CT	Agenesis of bilateral ICAs	Bilateral subcortical and border-zone regions/Prolonged MTT, TTP; Reduced CBF, CBV	Left cavernous region	PcomA aneurysm	Risk-factor control	3 years/mild headache
2	Female	49	Headache, nausea and vomit	CT	Right ICA agenesis, left ICA, and carotid canal hypoplasia	Left hemisphere/Prolonged MTT, TTP; Normal CBF, CBV	Between left ophthalmic artery and ECA branches	PcomA aneurysm	Endovascular embolization and STA-MCA bypass	13 months/unremarkable
3	Female	33	Hypomnesis	MRI	Agenesis of bilateral ICAs and unilateral VA	Right hemisphere and bilateral subcortical regions/Prolonged MTT, TTP; Reduced CBF, CBV	Between ICA and ECA, bilaterally	Dural arteriovenous fistula	Endovascular embolization with Onyx™ LES^a^	2 years/unremarkable
4	Female	46	Dizziness	CTA	Unilateral agenesis and fenestrated VA	Unreported	Between ECA branches and the distal end of right ICA	Basilar aneurysm	Radiographic follow-up	3 months/unremarkable
5	Female	65	Dizziness	MRI	Agenesis of bilateral ICA and elongation of bilateral VA	Unreported	Proximal terminus of the right ICA	Rete aneurysm	Radiographic follow-up	6 months/intermittent dizziness

^a^ Onyx™ Liquid Embolic System 18 (Covidien PLC). CT, computed tomography; CTA, computed tomography angiography; CBF, cerebral blood flow; CBV, cerebral blood volume; DSA, Digital subtraction angiography; ECA, external carotid artery; ICA, internal carotid artery; MCA, middle cerebral artery; MRI, magnetic resonance imaging; MTT, mean transit time; PcomA, posterior communicating artery; SAH, subarachnoid hemorrhage; STA, superficial temporal artery; VA, vertebral artery; TTP, time to peak.

Complicated cerebrovascular disorders were seen in all patients. Three patients had intracranial aneurysms, of which two were located in the PCA, with PcomA providing collateral circulation to the ICA. One of them suffering SAH finally achieved a good outcome with bypass surgery plus endovascular treatment. In addition, one case was diagnosed DAVF and the fistula was completely obliterated by embolization with Onyx™ LES. Another patient obtained rete aneurysm.

## Discussion

ICA agenesis is often associated with concomitant vascular lesions (1). As the flow stress to collateral arteries and the insufficiency of collateral circulation, altered hemodynamics might disrupt cerebrovascular homeostasis. The present case series suggested that ICA agenesis is the potential risk factor for cerebrovascular disorders such as intracranial or rete aneurysms and carotid rete mirabile.

The possible pathogenesis is that the increased hemodynamic stress secondary to ICA agenesis continuously acts on the collateral circulation, mostly provided by the basilar artery or posterior or anterior communicating artery, and increases the risk of abnormal enlargement or elongation of these collateral arteries. Our results supported the previous finding that patients with ICA agenesis were more susceptible to intracranial aneurysms (24%–34%) than the normal subjects (2%–4%) (6). The proportion of intracranial aneurysms in our study is even higher than reported and they were all located in the blood compensatory artery. Elazab et al. reported a complicated basilar artery aneurysm in a 2-month-old girl with ICA agenesis, demonstrating that intracranial aneurysms can also appear in the infant with ICA agenesis (7).

Besides the pressure for blood compensatory arteries from posterior and contralateral circulation, the rete mirabile was usually developed as the compensation from extracranial arteries, presented by the collateral plexus between the ECA branches and the ICA. In our study, six of the 8 ICAs with agenesis were accompanied by carotid rete mirabile. This phenomenon is considered to be a vascular response to the late regression of the ICA resulting from late embryological insult (8). Due to the late ICA regression, most of the embryonic vessels have already become permanent, and then rete ensues as a vascular response and compensatory circulation.

The changes in CT perfusion provided evidence for the abnormal hemodynamic pattern. Mikami et al. (9). observed reduced CBF in patients with ICA agenesis. In our cases, two patients had the hemodynamic compromise of prolonged MTT and TTP with reduced CBV and CBF, and one had mildly impaired hemodynamics of prolonged MTT and TTP with normal CBV and CBF. The impaired perfusion was mainly noted in the subcortical or border-zone region. Although hemodynamic insult was apparent and increased the risk for cerebrovascular disorders, rare cases reported in pediatric patients imply that collateral compensation is sufficient to support intracranial perfusion (10, 11).

Therefore, most patients with ICA agenesis could be asymptomatic due to sufficient collateral compensation. While asymptomatic ICA agenesis was few reported because these lesions might not be noticed until diagnosed incidentally during workup for other abnormalities associated with carotid artery agenesis or at autopsy (12). For example, they can be detected alongside the examination for asymptomatic carotid rete mirabile (13, 14). Despite they were asymptomatic, ICA agenesis still is a predisposing factor for cerebrovascular disease (12). The intracranial aneurysms could be detected in further follow-up (15).

Patients who underwent CTA or DSA screen in the hospital usually have chief complaints. As a result, symptomatic cases were more likely to be detected and reported, presented mainly with SAH, ischemic stroke, or nerve palsy (16–18). All five enrolled patients (out of the 5,850 screened patients) were symptomatic. Our study showed that SAH can result not only from the rupture of an intracranial aneurysm but also from a rete aneurysm (19). It also has been reported that a contralateral ICA aneurysm ruptured into the ethmoid and sphenoid sinuses, and leads to intractable pulsatile epistaxis and hypovolemic shock. Therefore, radiographic surveillance for rete aneurysms and contralateral artery are warranted. The etiologies of DAVF in ICA anomalies included brain hypoperfusion, trauma, and venous hypertension (20), in which insufficient blood flow compensated by a rete network could be the main reason for DAVF in our patient. Furthermore, the presence of stenosis of the collateral arteries can facilitate the occurrence of stroke, and this could be aggravated by secondary insults from acquired risk factors, especially atherosclerosis. Other symptoms resulting from compression of enlarged collateral arteries or aneurysms have also been reported, including cranial nerve palsy, trigeminal neuralgia, and Horner syndrome (17, 21). Taken together, the control of risk factors is needed for these patients.

The abnormal angioarchitecture of ICA anomalies makes the treatment of complicated vascular disorders challenging. Effective measures for the treatment of ICA agenesis remain limited. Regular radiographic surveillance and risk factor control had been recommended (22). Surgical intervention should be carefully planned because of the special angioarchitecture. Failure to preserve or reconstruct these collateral arteries can result in devastating neurological sequelae. In case 2, bypass surgery was first performed to ensure sufficient intracranial perfusion in the event of occlusion of PcomA. Then the aneurysm was treated with endovascular treatment.

Our study has limitations. Firstly, the findings in this study were based on retrospectively collected data with a limited sample size. This solely imaging study was not adequate to clarify the complex pathophysiological process of ICA agenesis. Secondly, since all the patients enrolled in this study were symptomatic, we were difficult to robustly prove our statement in asymptomatic patients or normal individuals. Thirdly, long-time surveillance for the ICA anomalies and concomitant cerebrovascular disorders is limited. The lifetime risk of ICA anomalies needs further exploration.

## Conclusions

DSA findings in patients with ICA agenesis demonstrated various complicated abnormalities, including intracranial aneurysm, rete aneurysm, and carotid rete mirabile. Although there is a mild hemodynamic compromise in some cases, collateral arteries are sufficient to provide intracranial perfusion. However, after the first insult in the late embryological stage, the special angioarchitecture makes the inherent hemodynamic pattern vulnerable and predisposes patients to extrinsic risk factors.

## Data Availability

The original contributions presented in the study are included in the article/Supplementary Material, further inquiries can be directed to the corresponding author/s.
